# Draft genome of *Bacillus subtilis* strain YB955, prophage-cured derivative of strain 168

**DOI:** 10.1128/mra.00263-24

**Published:** 2024-07-22

**Authors:** Ryan King Perez, Jocelyn Sofia Chavez Rios, Jessica Grifaldo, Kurt Regner, Mario Pedraza-Reyes, Eduardo A. Robleto

**Affiliations:** 1 Department of Life Sciences, University of Nevada, Las Vegas, Nevada, USA; 2 Department of Biology, Division of Natural and Exact Sciences, University of Guanajuato, Guanajuato, Mexico; DOE Joint Genome Institute, Berkeley, California, USA

**Keywords:** genomes, *Bacillus subtilis*

## Abstract

We report the genome sequence of *Bacillus subtilis* strain YB955, a prophage-cured strain used as a model in DNA repair, bacterial physiology, and mutagenesis studies. The assembled and annotated draft genome contains 4,031 coding genes, 5 rRNAs, and 73 tRNAs. Compared to 168, YB955 has a 134,402 bp deletion.

## ANNOUNCEMENT

*Bacillus subtilis* strain YB955 has been used in the study of stationary-phase mutagenesis and bacterial physiology processes (1 – 13). YB955 is a SP-beta prophage-cured strain (14) originating from *B. subtilis* strain 168 (with 100% identity and 0 *e* value, CP136402) using DNA-mediated transformation and contains base substitutions conferring auxotrophy in histidine (*hisC952*), methionine (*metB5*), and leucine (*leuC427*) biosynthesis (15, 16). This project was conducted to compare the YB955 genome to the 168 strain, as there was no sequence available for YB955.

The strain YB955 was obtained from a culture collection stored at −80°C in the Robleto Lab at the University of Nevada, Las Vegas. It was grown aerobically (250 RPM, 37°C) in a defined minimal medium (17) until 90 minutes past the onset of stationary-phase growth. Genomic DNA was isolated (Promega Wizard Genomic DNA Purification Kit) from a single colony grown overnight in lysogeny broth, and the library was prepared using the Illumina DNA Prep Tagmentation kit. Sequencing was conducted using the Illumina NextSeq2000 platform (300-cycle flow) to yield 2 × 150 bp paired reads with the reported coverage of 67.6×. A 1%–2% PhiX control was spiked during the run to support optimal base calling. Read demultiplexing, trimming, and run analytics were performed using DRAGEN (v.3.10.12) (18).

**Fig 1:**
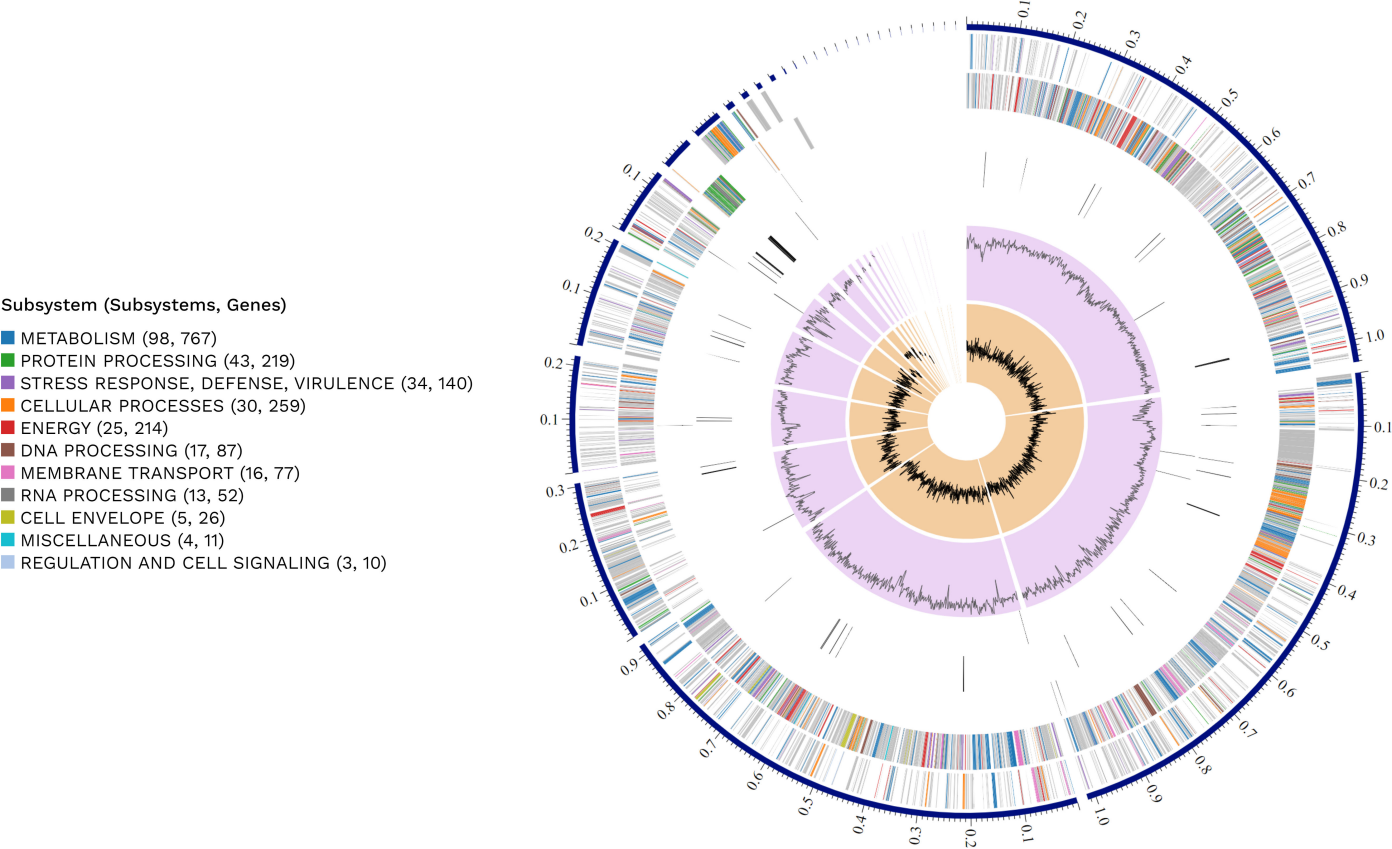
The genome of YB955 and its distribution of genomic annotations were visualized as a circular graphical display using PATRIC (v.3.26.4). Working inward from the exterior ring the figure presents the contigs, forward strand CDS, reverse strand CDS, RNA genes, CDS with homology to known antimicrobial resistance genes, CDS with homology to known virulence factors, GC content, and GC skew. The colors of the CDS on the forward and reverse strands indicate the subsystem that these genes belong to as seen in the subsystems key.

The raw sequence data underwent initial quality control with Trimmomatic (v.0.39) (19), and inherent read errors were removed utilizing the SPAdes algorithm (20). The refined sequences were assembled into contiguous sequences (contigs) employing the combined functionality of SPAdes and Unicycler (v.0.4.4) (21). Alignment of the reads back to the constructed contigs was executed with Bowtie2 and SAMtools (22, 23), and genome polishing was conducted using Pilon (v.1.24) (24). Annotation was performed using National Center for Biotechnology Information Prokaryotic Genome Annotation Pipeline (25), while RAST (v.2.0) (26) was utilized for functional categorization. Visualization of the genome was done using PATRIC (v.3.26.4) (27) through BV-BRC (v.3.34.11) (28) (Fig. 1). All tools were run with default parameters unless otherwise specified. The genome of YB955 is 4,014,474 bp long across 36 contigs. It has a GC content of 43.67% and an *N*_50_ of 1,015,588 (Table 1). The draft genome contains a total of 4,179 predicted genes and includes 4,096 CDS, 5 rRNAs, and 73 tRNAs. A total of 3,604 proteins with functional assignments were annotated, with 776 being assigned to a pathway. The YB955 strain showed a 56.26% AT content compared to that of the WT-168 strain, at 56.33%.

**TABLE 1:** Assembly details generated using values from the Prokaryotic Genome Annotation Pipeline and comprehensive genome analysis service after submission of the assembled genome^*a*^

Parameters	Value
Genome length	4,014,474
Contig N50	1,015,588
Contigs	36
GC content (%)	43.76
Plasmids	0

^*a*^ The assembled genome of YB955 had a total genome length of 4,014,474 bp and an average GC content of 43.76%.

Sequence comparison between 168 and YB955 yielded a 134,402-bp deletion, which starts at the gene *sprB* and ends with the gene *sprA* in 168 (coordinates: 2,152,086 → 2,286,408) (29). This size and the sequence of the deletion correspond to that of the SP-beta prophage (14). Overall, the analysis of the YB955 strain of *Bacillus subtilis* is important for the understanding of potential genetic factors influencing adaptive mutagenesis in bacteria.

## Data Availability

JAYFEV000000000. The version described in this paper is version JAYFEV010000000 The draft genome project data have been submitted under BioProject accession number PRJNA1054176 and Sequence Read Archive (SRA) accession number SRX24143230.
